# Treatment Patterns, Clinical Outcomes and Healthcare Costs of Advanced Non-Small Cell Lung Cancer: A Real-World Evaluation in Italy

**DOI:** 10.3390/cancers13153809

**Published:** 2021-07-29

**Authors:** Matteo Franchi, Diego Cortinovis, Giovanni Corrao

**Affiliations:** 1 National Centre for Healthcare Research and Pharmacoepidemiology, 20126 Milan, Italy; giovanni.corrao@unimib.it; 2 Laboratory of Healthcare Research & Pharmacoepidemiology, Department of Statistics and Quantitative Methods, University of Milano-Bicocca, 20126 Milan, Italy; 3 Medical Oncology, Asst H S Gerardo Monza, 20900 Monza, Italy; d.cortinovis@asst-monza.it

**Keywords:** non-small cell lung cancer, target therapy, immunotherapy, clinical practice, real-world, clinical outcomes

## Abstract

**Simple Summary:**

Targeted and immunotherapy have changed the treatment paradigm of NSCLC. We aimed at evaluating treatment patterns and real-world outcomes, including time-to-treatment failure, time-to-next-treatment, overall survival and healthcare costs, of advanced NSCLC patients in the era of immune-oncology therapies. Our results were generally coherent with those reported in other real-world studies, and they added novel evidence about the economic impact of such therapies in a large and unselected cohort of NSCLC patients treated in daily clinical practice.

**Abstract:**

We aimed at describing treatment pathways, clinical outcomes and healthcare costs of advanced non-small cell lung cancer (NSCLC) patients in Lombardy Region, Italy. Using healthcare administrative data, 37,562 patients with a new diagnosis of lung cancer between 2012 and 2019 were identified. Among these, patients who started a first-line treatment for advanced NSCLC with either pembrolizumab (*n* = 660) or tyrosine-kinase inhibitors (TKI) (*n* = 1245) before 30 June 2020 were included in the study cohort and followed-up until 31 December 2020. Among pembrolizumab users, median time-to-treatment failure (TTF) and median overall survival (OS) were 3.2 months and 13.6 months, respectively. About one third (34.1%) switched to second-line treatment (chemotherapy for all of them). Among TKI users, median TTF and median OS were 9.3 months and 18.4 months, respectively, and 37.1% of patients started second-line treatment (17.8% with TKI and 19.2% with chemotherapy). Average per-patient cumulative healthcare costs during the first year after first-line treatment start were 51,735 € and 30,708 €, respectively, in pembrolizumab and TKI first-line users. These results are coherent with those reported from other real--world studies and may help both clinicians and health decision makers.

## 1. Introduction

In Italy, lung cancer represents the third most common neoplasm and the leading cause of cancer mortality [1]. Non-small cell lung cancer (NSCLC) is the most common sub-type of lung cancer, accounting for about 85% of diagnosed lung cancers [2] and advanced disease at cancer diagnosis is detected in about 50% of patients [3]. The 5-year overall survival (OS) of advanced NSCLC is poor, varying from 26% in stage IIIB to 1% in stage IVB [4].

In the last decade, great insights into oncogenic alterations that lead to NSCLC development allowed to identify predictive and prognostic biomarkers and to develop target therapies, which were shown to be associated to clinical benefits, as compared to systemic chemotherapy, in patients with specific oncogene addicted NSCLCs [5]. In addition, immune checkpoint inhibitors (ICIs), namely pembrolizumab in first-line setting, was developed and is currently recommended for patients with tumor PDL1 high expression (≥50%) [6]. Based on these therapy developments, current Italian treatment guidelines recommend testing for mutations (EGFR, ALK, ROS-1, BRAF-V600) and PD-L1 biomarkers, in order to identify those patients who are eligible for targeted/immune checkpoint therapies [7]. Even though the efficacy of these treatments has been well documented by randomized clinical trials (RCTs) [8,9], few studies assessed the real-world treatment patterns and clinical outcomes of these therapies in large and unselected cohorts, especially in the era of immune-oncology therapies [10,11]. The current retrospective population-based cohort study is aimed at examining therapeutic pathways (including target therapies and ICIs), clinical outcomes and healthcare costs of advanced NSCLC patients in Lombardy Region, Italy.

## 2. Materials and Methods

### 2.1. Data Source

Administrative databases of Lombardy Region, a Northern Italy region accounting for more than 10 million inhabitants, were used. In Lombardy, management of the National Health Service (NHS) has been associated since 1997 with an automated system of databases to collect health information such as (i) demographic and administrative data on NHS beneficiaries, including information on the date of entry (birth or immigration) and exit (death or emigration) during the entire time window available; (ii) hospital discharge records reporting information on inpatient primary diagnosis, up to five coexisting conditions and procedures coded according to the International Classification of Diseases, 9th Revision, Clinical Modification (ICD-9-CM) classification system; (iii) drugs dispensed by territorial pharmacies and medicines directly administered in the outpatient setting and day-hospital coded according to the Anatomical Therapeutic Chemical (ATC) classification system; (iv) data on outpatient services, including specialist visits, laboratory tests and diagnostic imaging. Record linkage between databases was performed by means of an identification code assigned to each NHS beneficiary. In order to preserve the privacy of the beneficiaries, identification codes were de-identified and the conversion table was deleted. 

Specific diagnostic and therapeutic codes used for the current study are given in Supplementary Materials (Table S1).

### 2.2. Target Population

All individuals who during the years 2012 to 2019 were resident in Lombardy and beneficiaries of the NHS and had at least one hospital admission with a diagnostic code of lung cancer were selected. The first hospitalization for lung cancer was labelled “index hospitalization”. Among these, were excluded patients (i) beneficiaries of the NHS for less than 5 years before index hospitalization, (ii) with a diagnostic code of any malignant neoplasm and/or an antineoplastic treatment in the 5 years prior to the index hospitalization (i.e., for excluding prevalent cancer cases and/or multiple cancers), (iii) aged less than 18 years at index hospitalization, (iv) died during index hospitalization, (v) with data inconsistences/errors.

### 2.3. First-Line Treatment for Advanced NSCLC

Since ICD-9-CM diagnostic codes do not allow distinguish cancer sub-types (i.e., NSCLC vs. other thoracic cancer types) and the stage of the disease (i.e., locally advanced/metastatic cancer) is not recorded in our databases, we could not identify advanced NSCLC patients based on a histopathological analysis. Conversely, we used information on their systemic treatments as a surrogate of their cancer histology. Indeed, as indicated by the Italian guidelines on lung cancer, first-line systemic treatments for advanced NSCLC include conventional chemotherapy (in squamous non-oncogene addicted cancers), tyrosine-kinase inhibitors (TKI, in oncogene addicted cancers), pembrolizumab alone (in non-oncogene addicted cancers with expression of PD-L1 ≥ 50%) or pembrolizumab in association with chemotherapy (in non-squamous non-oncogene addicted cancers with expression of PD-L1 < 50%) [7]. Except for chemotherapy, that represents a backbone treatment also for SCLC, TKIs and pembrolizumab are specifically approved for advanced NSCLC. Thus, we included in our analyses only patients who started a first-line treatment with TKIs or pembrolizumab. Since administration of pembrolizumab in association with chemotherapy was approved in Italy only in December 2019 for non-squamous NSCLC and in December 2020 for squamous NSCLC, this therapeutic option was not considered in the current study.

Operatively, during the period from index hospitalization to 30 June 2020, all patients with a new prescription of either TKI or pembrolizumab were identified. According to the drug prescribed first, patients were defined as exposed to first-line TKI or first-line pembrolizumab, and the date of the first prescription was labelled index date. In order to exclude the few patients treated with pembrolizumab in association with chemotherapy, those with evidence of chemotherapy in the 21 days (i.e., the duration of a chemotherapy cycle [7,11]) after index date were excluded. Finally, since pembrolizumab is currently approved also as second-line treatment after failure of conventional chemotherapy, patients with evidence of chemotherapy in the six months before index date were further excluded. The remaining patients were included in the study cohort and followed-up until 31 December 2020.

### 2.4. Outcomes

Clinical outcomes included time-to-treatment failure (TTF), time-to-next treatment (TTNT) and overall-survival (OS). TTF, which represents a measure of treatment duration, was defined as the time between index date (i.e., the date of first-line treatment start) and the earliest date between treatment discontinuation for any cause (i.e., the outcome of interest), migration or end of data availability (i.e., 31 December 2020). TTNT was defined as the time between index date and the earliest date between starting a next line of treatment (i.e., the outcome of interest), death, migration or end of data availability. Finally, OS was defined as the time between index date and the earliest date between death for any cause (i.e., the outcome of interest), migration or end of data availability.

Moreover, an economic outcome was also assessed by measuring the average per-capita cumulative healthcare costs sustained by the NHS, including all inpatient and outpatient costs from index date to the earliest date between death, migration or end of data availability.

### 2.5. Statistical Analyses

Descriptive tables were used for summarizing baseline characteristics, including age at index date, sex, year of treatment start and surgery within 6 months from lung cancer diagnosis. TTF, TTNT and OS were estimated by using the Kaplan-Meier (KM) estimator. In the TTNT analysis, death was considered as competing event.

Cumulative healthcare costs according to first-line treatment were calculated by means of the Bang and Tsiatis estimator [12], a method that takes into account censored cost data. For each patient, cumulative healthcare cost were calculated by summing up direct costs sustained by the NHS for inpatient and outpatient services and drug dispensations supplied starting from first-line treatment start.

All analyses were performed using SAS 9.4 (Cary, NC, USA).

### 2.6. Secondary Analyses

In a secondary analysis, the number and baseline characteristics of patients treated with second- (or further) line ICIs were evaluated. In Italy, ICIs can be prescribed after failure of a chemotherapy-based first-line treatment [7]. Thus, all patients from the target population who started treatment with pembrolizumab, nivolumab and atezolizumab within 31 December 2020 were identified. In order to ensure that pembrolizumab was given as second-line treatment, only patients for which chemotherapy was administered within 6 months before the date of the first pembrolizumab administration were included in this analysis. Conversely, nivolumab and atezolizumab are only approved in Italy as second- (or further) line treatment options.

## 3. Results

During the period 2012–2019, 62,660 patients with diagnosis of lung cancer were identified, of which 37,562 met the inclusion criteria. The process of cohort selection is given in Supplementary Materials (Figure S1).

### 3.1. First-Line Tratment for Advanced NSCLC

Starting from index hospitalization (i.e., the first hospitalization with a diagnostic code of lung cancer), 1905 patients started a first-line treatment for advanced NSCLC with one of the therapeutic strategies considered in the current study, of which 660 with first-line pembrolizumab and 1245 patients with first-line TKI. Among the latter, 984 patients were treated with first/second generation EGFR inhibitors (639 with gefitinib, 175 with afatinib and 170 with erlotinib), 85 patients with third generation EGFR inhibitors (osimertinib), 101 patients with first generation ALK inhibitors (crizotinib), 67 patients with second generation ALK inhibitors (66 with alectinib and 1 with ceritinib) and 8 patients with BRAF inhibitors (dabrafenib + trametinib). 

Patient’s characteristics are shown in Table 1. Patients treated with pembrolizumab were mainly men (69.6%), had a median age at first-line treatment start of 69 years and about 19% underwent surgery within six months after lung cancer diagnosis. Few patients (13.2%) started treatment in 2017, since pembrolizumab alone was approved in Italy in August 2017. Conversely, patients treated with TKI were more frequently female (60.3%), with a median age at treatment start of 71 years, and about one fifth (20.7%) were surgically treated.

### 3.2. Time-to-Treatment Failure

Among pembrolizumab first-line users, during a median (mean) treatment duration of 3.2 (7.2) months, 567 (85.9%) episodes of discontinuation were observed. The cumulative probability of discontinuing treatment was 72.9%, 87.6% and 94.5%, respectively, after 1 year, 2 years and 3 years from treatment start.

**Figure 1:**
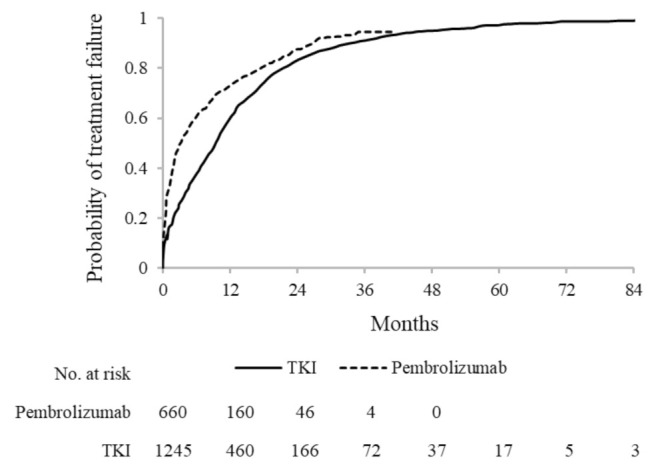
Kaplan-Meier estimates of time-to-treatment failure among 660 and 1245 advanced NSCLC patients treated, respectively, with first-line pembrolizumab and tyrosine-kinase inhibitors (TKI).

A median (mean) treatment duration of 9.3 (12.2) months was observed among TKI first-line users. Overall, 911 (70.4%) patients discontinued treatment during follow-up. The cumulative probability of discontinuing was 60.1%, 83.2%, 91.0% and 97.1% after 1 year, 2 years, 3 years and 5 years, respectively, from treatment start (Figure 1).

### 3.3. Time-to-Next Treatment and Second-Line Treatments

Among pembrolizumab first-line users, the number of patients who switched to next treatment was 225 (34.1%), and all of them switched to conventional chemotherapy. Cumulative incidence of starting next treatment was 28.2%, 35.5% and 37.9%, respectively, after 1 year, 2 years and 3 years from treatment start.

**Figure 2:**
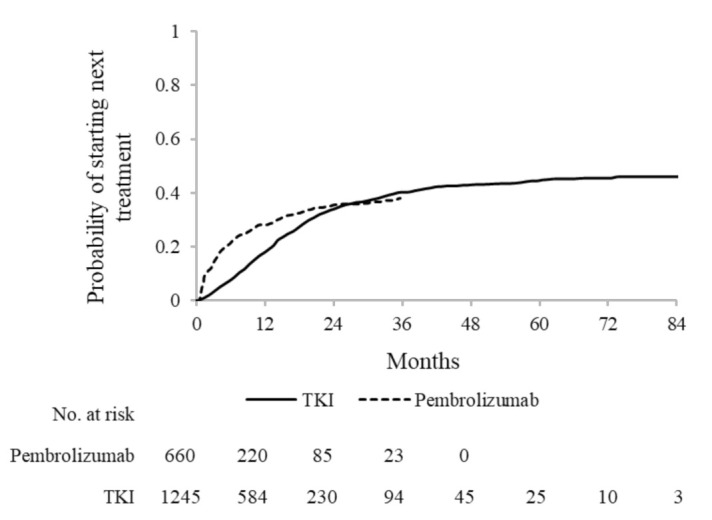
Kaplan-Meier estimates of time-to-next treatment among 660 and 1245 advanced NSCLC patients treated, respectively, with first-line pembrolizumab and tyrosine-kinase inhibitors (TKI).

Among TKI first-line users, 468 (37.6%) started next treatment, of which 224 (18.0%) started a TKI agent (different from the first-line one) and 242 (19.4%) started conventional CT. In particular, the third generation EGFR inhibitor osimertinib was the most frequent second-line TKI used in patients initially treated with first/second generation EGFR inhibitors (i.e., gefitinib, afatinib and erlotinib). Patients initially treated with crizotinib most frequently switched to second generation ALK inhibitors. All patients initially treated with osimertinib switched to traditional chemotherapy. Therapeutic patterns from first-line to second-line treatment are shown in Table 2. Cumulative incidence of starting next treatment was 17.9%, 33.6%, 40.1% and 44.4% after, respectively, 1 year, 2 years, 3 years and 5 years from treatment start (Figure 2). 

In the secondary analysis, 1720 patients were identified as starting second (or further) line treatment with ICIs. Of these, 150 patients were treated with pembrolizumab, 1244 patients with nivolumab and 326 patients with atezolizumab. Patient’s characteristics are shown in Table 3.

### 3.4. Overall Survival

During a median follow-up of 11.5 months, 396 (60.0%) deaths were observed among patients treated with pembrolizumab. The 1-year, 2-year and 3-year OS was 53.5%, 37.7% and 31.3%, respectively. Median OS was 13.6 months (95% CI: 11.7–17.2).

**Figure 3:**
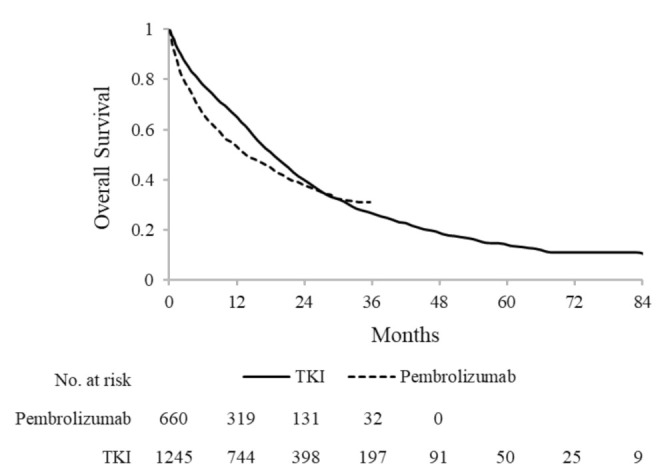
Kaplan-Meier estimates of overall survival among 660 and 1245 advanced NSCLC patients treated, respectively, with first-line pembrolizumab and tyrosine-kinase inhibitors (TKI).

Among patients treated with TKI, 908 (72.0%) deaths were observed during a median follow-up of 15.3 months. The 1-year, 2-year, 3-year and 5-years OS was 65.0%, 39.7%, 26.7% and 14.0%, respectively, with a median OS of 18.4 (95% CI: 16.8–19.9) (Figure 3). Median OS among 1069 patients treated with first-line EGFR inhibitors and among 176 patients treated with first-line ALK inhibitors was 17.6 months (95% CI: 16.2–19.1) and 26.9 months (95% CI: 22.1–39.9), respectively.

### 3.5. Cumulative Healthcare Costs

**Figure 4:**
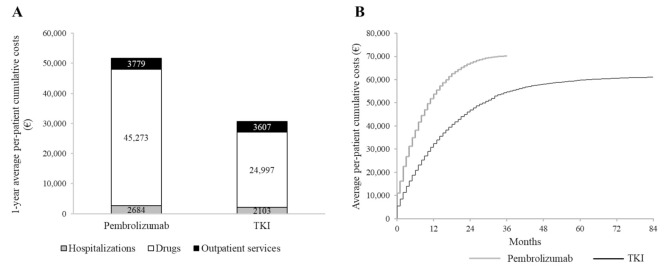
Average per-patient cumulative cost during the first year after first-line treatment start (**A**) or during the entire follow-up (**B**), among 660 and 1245 advanced NSCLC patients treated, respectively, with first-linepembrolizumab and tyrosine-kinase inhibitors (TKI).

Cumulative NHS healthcare costs according to first-line treatment are shown in Figure 4. On average, 51,736 € and 30,707 € were spent for each patient treated with first-line pembrolizumab and TKI, respectively, within the first 12 months after starting first-line therapy. The average cost of a patient on treatment with pembrolizumab included 2684 € for hospitalization, 3779 € for outpatients services and 45,273 € for drugs (of which 44,171 € for oncologic and ancillary therapies). Corresponding figures for patients on treatment with TKI were 2103 €, 3607 € and 24,997 € (24,055 €), respectively (Figure 4A). In Supplementary Materials (Table S2) is detailed the distribution of outpatients services dispensed to cohort patients during the observation period. Cumulative healthcare costs during the entire follow-up are shown in Figure 4B. In particular, total healthcare costs were 66,583 € and 70,178 €, respectively, 2 years and 3 years after starting pembrolizumab treatment, and 46,000 € and 54,299 €, respectively, after starting TKI treatment.

## 4. Discussion

The current study provided a depiction of real-world treatment patterns and outcomes of advanced NSCLC in a large and unselected population-based cohort of patients with lung cancer, reflecting the daily clinical practice in Lombardy, the most populated Italian region.

Our results are coherent with those reported from other observational studies. In our cohort, the median OS of patients treated with first-line TKI was 18.4 months irrespectively of specific oncogene addiction, very similar to the median OS of 18.8 months observed in mutation-positive advanced NSCLC patients included in a German retrospective analysis [11]. When considering patients treated with first-line ALK inhibitors, the median OS (i.e., 26.9 months) was very similar to the median OS of 581 patients treated with first-line ALK inhibitors in a retrospective cohort study in the US (i.e., 25.8 months) [13]. Among patients who progressed after first-line TKI, osimertinib was the most frequent agent used as second-line therapy. This data is consistent with national guidelines, which recommend the use of osimertinib as second-line treatment of patients with disease progression after first-line TKI inhibitors, as well as with real-world studies, which showed that osimertinib is highly effective in patients who received prior EGFR inhibitors [14,15].

Among patients treated with first-line pembrolizumab, we observed a median TTF of 3.2 months. In a real-world analysis on 524 stage IV NSCLC patients prescribed with first-line pembrolizumab treatment in community and academic cancer clinics across the USA, the median duration of treatment was 6.9 months and 2.3 months, respectively, in 386 patients with ECOG performance status (PS) comprised between 0 and 1, and in 138 patients with ECOG PS equal to 2. Moreover, the Kaplan-Meier estimates of the cumulative probability of discontinuing pembrolizumab after 12 months from treatment start was 63.6% and 83.8%, respectively, in patients with ECOG performance status 0-1 and 2 [16]. The latter values are very similar to that observed in our cohort, i.e., 72.9%, irrespective of the ECOG performance status, which was not available in our databases. A multicentre study conducted in Canada on 190 advanced NSCLC treated with pembrolizumab as first or subsequent line of therapy showed a median duration of treatment of 4.4 months [17]. Moreover, an observational study conducted in the US showed a median TTF of 3.5 in 907 advanced NSCLC patients treated with first-line pembrolizumab or nivolumab in monotherapy and a median TTF of 3.0 months in 324 patients treated with first-line pembrolizumab in association with carboplatin and pemetrexed [10]. 

In our cohort, the median OS of patients treated with first-line pembrolizumab, i.e., 13.6 months, was lower than that observed in other real-world studies. In particular, in three observational studies carried out in the US, median OS ranged from 18.9 months to 25.6 months [10,17,18]. Notably, median OS varied substantially between patients with ECOG PS 0-1 and those with ECOG PS 2 (16.7 months vs. 5.8 months in advanced NSCLC patients treated with first or subsequent line of therapy) [17]. Moreover, a multicentre observational study conducted in Japan on 213 advanced NSCLC patients treated with first-line pembrolizumab showed a median OS of 17.8 months [19]. The differences observed between our study and other real-world studies may be due to the population-based nature of our study, which included all beneficiaries of the NHS treated with first-line pembrolizumab in Lombardy, reflecting the potential heterogeneity of patients’ characteristics and healthcare facilities. Even though current guidelines do not directly address the issue of pembrolizumab administration in the first-line setting for patients with worst clinical profile (i.e., the elderly, those with frailty or those with ECOG PS >1), pembrolizumab may represent a valid option still in the lack of supporting evidence [20]. A multicentre retrospective Italian observational study evaluating survival in 153 patients with ECOG PS 2 treated with first-line pembrolizumab showed a very poor median OS (i.e., 3.0 months) [21]. Thus, this and other characteristics that we could not measure with our data may have influenced the median OS observed in our cohort.

The economic analysis showed that costs sustained by the NHS for treatment of advanced NSCLC were mainly driven by oncology therapies, followed by outpatient health service and hospitalizations. This pattern is coherent with that observed in an observational cohort study conducted in Italy on 191 NSCLC patients in the pre-immunotherapy era [22].

The current study has some limitations. First, since our data did not allow the direct identification of NSCLC patients (because diagnostic ICD-9-CM codes does not distinguish between cancer sub-types), we do not know the target population of NSCLC patients from which patients included in our cohort generated from. Thus, we could not estimate the percentage of patients who started a first-line systemic treatment, among all patients diagnosed with advanced NSCLC. This represents one of the major limitations of our data, which are not linkable with other data sources (for example, with data from pathological anatomy), thus making not possible to characterize patients based on their histological diagnosis. Second, since information on cancer stage was not available in our databases, we were not able to identify patients with upfront metastatic cancer and those with distant recurrence. Even though diagnostic ICD-9-CM codes allows identifying distant metastasis, our data only refer to inpatient diagnosis. Thus, diagnoses carried out in the outpatient setting are not available. Third, we could not identify and distinguish between conventional chemotherapy drugs (e.g., cisplatin/carboplatin/gemcitabine/vinorelbine/taxanes), because they are generally recorded in our databases without indication of the ATC code (conversely, high-cost or innovative oncologic drugs administered in day-hospital or in outpatient setting, such as targeted therapies and ICIs considered in the current study, are recorded with the corresponding ATC code). For this reason, we could not focus on treatment patterns of patients treated with first-line chemotherapy, which represents a considerable portion of all advanced NSCLC starting a first-line systemic treatment [10]. Fourth, treatments administered within RCTs are not recorded in our databases, because they are directly paid by the study sponsor. 

To our knowledge, this represents the first study assessing pharmacoutilization, clinical and economic outcomes, conducted in Italy on a large-scale population. The population-based nature of the study guarantees the representativeness of routine clinical practice in Lombardy and the generalizability of the results and reflects the potential heterogeneity in the management of lung cancer. Indeed, the study cohort included all the potential NHS beneficiaries who had a new diagnosis of lung cancer during the recruitment period, with no restriction on clinical or toxicity profile.

## 5. Conclusions

In conclusion, these results may be of great interest for clinicians, in order to increase their knowledge about clinical outcomes of these patients, and for health decision makers, providing them with information about the economic impact of such therapies.

## Figures and Tables

**Table 1:** Baseline characteristics of 660 advanced NSCLC patients treated with first-line pembrolizumab and 1245 patients treated with first-line TKI.

	First-Line Treatment	
Characteristics	Pembrolizumab *n* = 660	TKI *n* = 1245
Sex		
Male	459 (69.6)	494 (39.7)
Female	201 (30.4)	751 (60.3)
Age ^¥^, median (min-max)	69 (41–91)	71 (29–93)
<60	96 (14.5)	272 (21.8)
60–69	212 (32.1)	275 (22.1)
70–79	281 (42.6)	487 (39.1)
≥80	71 (10.8)	211 (17.0)
Year of First-Line Treatment Onset		
2012	0	64 (5.1)
2013	0	93 (7.5)
2014	0	132 (10.6)
2015	0	161 (12.9)
2016	0	140 (11.2)
2017	87 (13.2)	213 (17.1)
2018	250 (37.9)	215 (17.3)
2019	266 (40.3)	140 (11.2)
2020 ^§^	57 (8.6)	87 (7.0)
Surgery ^†^		
No	534 (80.9)	987 (79.3)
Yes	126 (19.1)	258 (20.7)

^¥^ Calculated at index date; ^§^ Up to 30 June 2020; ^†^ Within six months after lung cancer diagnosis.

**Table 2:** Treatment switch among 1245 advanced NSCLC patients treated with first-line TKI.

First-Line Treatment	Second-Line Treatment		
	TKI	CT	Total
Gefitinib *n* = 639	133 (111 osimertinib, 18 erlotinib, 2 afatinib, 1 crizotinib, 1 nintedanib)	144	277
Afatinib *n* = 175	42 (29 osimertinib, 12 gefitinib, 1 erlo- tinib)	32	75
Erlotinib *n* = 170	25 (21 osimertinib, 4 gefitinib)	43	69
Crizotinib *n* = 101	24 (17 alectinib, 7 ceritinib)	8	32
Osimertinib *n* = 85		12	12
Alectinib *n* = 66		3	3
Dabrafenib + trametinib *n* = 8			0
Ceritinib *n* = 1			0
Total (%) *n* = 1245	224 (18.0%)	242 (19.4%)	468 ^§^ (37.6%)

^§^ Two patients started second-line treatment with nivolumab.

**Table 3:** Baseline characteristics of advanced NSCLC patients treated with second/further-line immune checkpoint inhibitors (ICIs).

	Second-(or Further) Line Treatment		
	Pembrolizumab *n* = 150	Nivolumab *n* = 1244	Atezolizumab *n* = 326
Sex			
Male	102 (68.0)	879 (70.7)	213 (65.3)
Female	48 (32.0)	365 (29.3)	113 (34.7)
Age ^¥^, median (min-max)	68 (34-85)	70 (39-89)	70 (41-89)
<60	30 (20.0)	197 (15.8)	54 (16.6)
60–69	48 (32.0)	415 (33.4)	102 (31.3)
70–79	58 (38.7)	514 (41.3)	131 (40.2)
≥80	14 (9.3)	118 (9.5)	39 (11.9)
Year of Second-LineTreatment			
2015	0 (0)	2 (0.2)	0 (0)
2016	0 (0)	140 (11.3)	0 (0)
2017	29 (19.3)	429 (34.5)	0 (0)
2018	44 (29.3)	325 (26.1)	27 (8.3)
2019	36 (24.0)	203 (16.3)	176 (54.0)
2020	41 (27.3)	145 (11.7)	123 (37.7)
Surgery ^†^			
No	116 (77.3)	965 (77.6)	244 (74.9)
Yes	34 (22.7)	279 (22.4)	82 (25.1)

^¥^ At ICI treatment start; ^†^ Within six months after lung cancer diagnosis.

## Data Availability

The data that support the findings of this study are available from Lombardy Region, but restrictions apply to the availability of these data, which were used under license for the current study, and so are not publicly available. Data are however available from the Lombardy Region upon reasonable request.

## Supplementary Materials

The following are available online at https://www.mdpi.com/article/10.3390/cancers13153809/s1, Table S1: ICD-9-CM codes of diagnosis/procedures, ATC codes of drugs and Regional codes of outpatients services used for the current study. Table S2: Distribution of outpatient services dispensed to 660 advanced NSCLC patients treated with first-line pembrolizumab and to 1,245 patients treated with first-line TKI. Figure S1: Flow-chart of cohort selection.
