# Supplementary material for: Treatment Patterns, Clinical Outcomes and Healthcare Costs of Advanced Non-Small Cell Lung Cancer: A Real-World Evaluation in Italy
Source: Cancers (Basel). 2021 Jul 29;13(15):3809. doi: 10.3390/cancers13153809 (PMC8345176; doi:10.3390/cancers13153809)

Tables S1. ICD-9-CM codes of diagnosis/procedures, ATC codes of drugs and Regional codes of outpatients services used for the current study.

Diagnosis (ICD-9-CM codes)	
Lung cancer	162* (except 162.0)
Chemotherapy	V58.1
Procedures (ICD-9-CM codes)	
Lung surgery	
Other local excision or destruction of lesion or tissue of lung	32.29
Segmental resection	32.3
Lobectomy	32.4
Complete pneumonectomy	32.5
Radical dissection of thoracic structures	32.6
Other excision of lung	32.9
Chemotherapy/antineoplastic biological agents	99.25, 99.28
Drugs (ATC codes)	
Immunotherapy	
Pembrolizumab	L01XC18
Nivolumab	L01XC17
Atezolizumab	L01XC32
Tyrosine kinase inhibitors (TKI)	
Erlotinib	L01XE03
Gefitinib	L01XE02
Afatinib	L01XE13
Osimertinib	L01EB04
Crizotinib	L01ED01
Alectinib	L01ED03
Trametinib	L01EE01
Dabrafenib	L01EC02
Chemotherapy	
Cisplatin	L01XA01
Carboplatin	L01XA02
Docetaxel	L01CD02

Pemetrexed	L01BA04
Paclitaxel	L01CD01
Vinorelbine	L01CA04
Gemcitabine	L01BC05
**Outpatients service (Regional codes)**	
Chemotherapy with administration of high-cost drugs (file F 5 e 3)	MAC01
Chemotherapy without administration of high-cost drugs (file F 5 e 3)	MAC02
Anticancer therapy with administration of oral drug or IM	MAC04
Injection or infusion of chemotherapy drugs	99.25

Tables S2. Distribution of outpatient services dispensed to 660 advanced NSCLC patients treated with first-line pembrolizumab and to 1,245 patients treated with first-line TKI.

	First-line treatment	
	Pembrolizumab	TKI
Laboratory exams	69.3%	71.8%
Diagnostic imaging	10.5%	9.9%
Specialist visits	8.9%	8.9%
Other	11.3%	9.5%

Figure S1. Flow-chart of cohort selection.

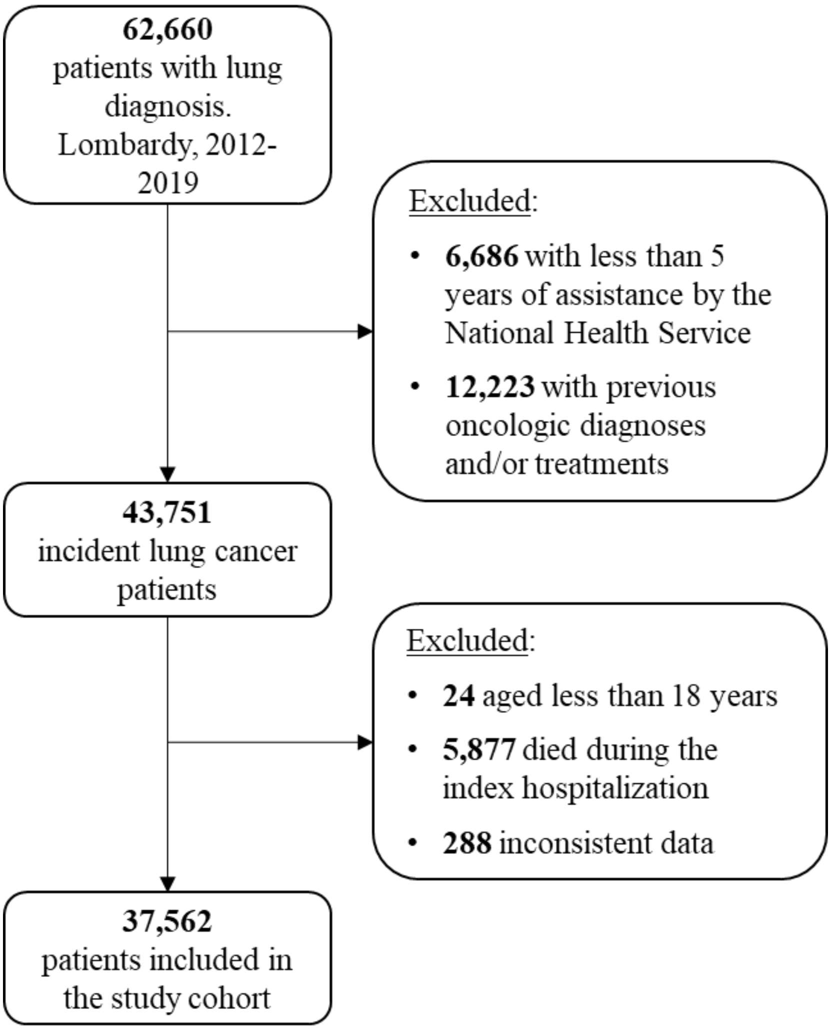

Supplement: Supplementary file 1 [file cancers-13-03809-s001.zip › cancers-1279710-supplementary.pdf]
